# [Fe(phen)_**3**_]^**2+**^ and [Fe(phen)_**3**_]^**2+**^-Loaded Nanostructured Lipid System: *In Silico, In Vitro,* and *In Vivo* Efficacy against *Mycobacterium tuberculosis*

**DOI:** 10.1021/acsomega.5c08350

**Published:** 2025-11-27

**Authors:** Fernanda Manaia Demarqui, Christian Shleider Carnero Canales, Rachel Temperani Amaral Machado, Rafael Miguel Sábio, Ingrid Gracielle M. Silva, Karine B. Barros-Cordeiro, Sônia N. Báo, Masanori Asai, Sandra M. Newton, Paul R. Langford, Fernando Rogério Pavan

**Affiliations:** † Tuberculosis Research Laboratory, School of Pharmaceutical Sciences, 28108 São Paulo State University − UNESP, Araraquara, São Paulo 14801-903, Brazil; ‡ Vicerrectorado de Investigación, Universidad Autónoma Del Perú, Lima 15842, Perú; § Microscopy and Microanalysis Laboratory, Department of Cell Biology, Institute of Biological Sciences, 28127 University of Brasília, Brasília, Federal District 70365-070, Brazil; ∥ Paediatric Infectious Diseases, Department of Infectious Disease—Faculty of Medicine, 156432 Imperial College, London SW7 2AZ, England

## Abstract

Tuberculosis (TB), caused by *Mycobacterium tuberculosis* (*Mtb*), remains one of the leading causes of mortality from infectious diseases worldwide. So, this study investigates the antimicrobial potential of [Fe­(phen)_3_]^2+^ (FEP) and FEP-loaded nanostructured lipid systems (NLS@FEP) as an innovative therapeutic approach for TB. The FEP showed promising antimycobacterial activity in simulated physiological environments, with minimum inhibitory concentrations (MIC_90_) from 3.92 to 0.98 μg mL^–1^. FEP combination with rifampicin or pretomanid significantly reduced the MIC_90_, with fractional inhibitory concentration index (FICI) of 0.27 and 0.103, respectively. Field emission scanning electron microscopy (FE-SEM) analysis revealed significant structural alterations in the *Mtb* cell wall, suggesting that FEP interferes with its synthesis. *In silico* analyses and whole-genome sequencing (WGS) supported these findings, identifying mutations in key genes, such as *ponA1*, which encodes a penicillin-binding protein involved in peptidoglycan synthesis. *In silico* modeling predicted high FEP affinity for PonA1, in line with FE-SEM observations; however, these predictions are hypothesis-generating and require functional validation. FEP-loaded nanostructured lipid system (NLS@FEP) was designed to optimize FEP activity, which improved its stability and bioavailability. In a murine model infected with *Mtb* H37Rv, free FEP and NLS@FEP achieved complete elimination of pulmonary infection.

## Graphical abstract

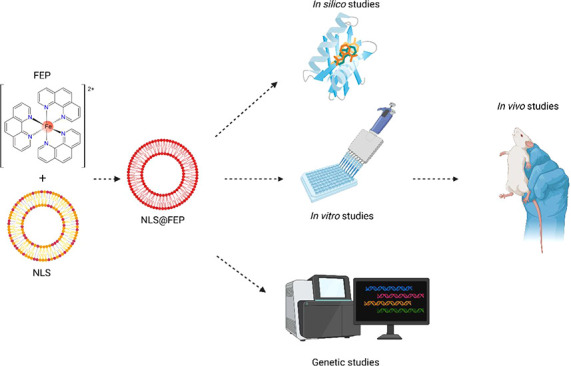

## 1 Introduction

Tuberculosis (TB), caused by *Mycobacterium tuberculosis* (*Mtb*), remains a major cause of mortality from infectious diseases. Despite advances in diagnosis and treatment, the emergence and spread of multidrug-resistant (MDR) and extensively drug-resistant (XDR) strains have undermined the efficacy of current regimens and created an urgent need for new therapeutic strategies.^1–3^ Conventional regimens, based on prolonged combinations of isoniazid (INH), rifampicin (RIF), ethambutol, and pyrazinamide, require at least six months of treatment and are associated with adverse effects that limit adherence and promote micobacterial resistance.^4^ In the case of drug-resistant TB, treatment duration can extend up to two years with second-line agents, which are often less effective and more toxic. In this context, innovative compounds capable of overcoming resistance mechanisms and improving bioavailability are highly desirable.^5^

The tris­(1,10-phenanthroline)­iron­(II) complex ([Fe­(phen)_3_]^2+^, FEP) has shown antimicrobial properties linked to disruption of cell-wall integrity and generation of reactive oxygen species. Its low cost and water solubility make it an attractive candidate for evaluation against *Mtb*. However, the limited stability and potential rapid metabolism of the free compound may compromise its therapeutic performance.^6,7^

Nanoplatforms, particularly nanostructured lipid systems (NLS), offer advantages for the encapsulation and controlled release of antimicrobial agents by enhancing stability, bioavailability, and safety.^8–11^ In this study, we evaluated the antimycobacterial potential of FEP and its encapsulated formulation (NLS@FEP) through an integrated approach combining *in silico* modeling, *in vitro* microbiological assays, and *in vivo* validation in a murine TB model.

## 2 Materials and Methods

### 2.1 Materials

Middlebrook 7H9 culture medium was obtained from Kasvi (Paraná, Brazil). Catalase (4,000 units mg^–1^) was purchased from Thermo Fisher Scientific Inc. (MA, USA). Bovine serum albumin (96%) was acquired from Interlab Confiança (São Paulo, Brazil). Roswell Park Memorial Institute (RPMI) 1640 medium was purchased from Gibco (Carlsbad, CA, USA). Cholesterol (99%), phosphatidylcholine, sodium oleate (99%), Eumulgin, resazurin (80%), glycerol (99%), polysorbate 80, rifampicin (RIF) (97%), isoniazid (INH) (99%), dextrose, gentamicin sulfate (Pharmaceutical Secondary Standard), amphotericin B (Certified Reference Material), sodium chloride (99.5%), and 1,10-phenanthroline (99%), as well as phosphate-buffered saline (PBS), glutaraldehyde (50%), paraformaldehyde (95%), potassium ferricyanide (99%), and osmium tetroxide (99.8%), were also purchased from Sigma-Aldrich (St. Louis, MO, USA).

### 2.2 *Mtb* Antimicrobial Susceptibility at Different Physiological NaCl Concentrations

The influence of NaCl (14.5 mM, 125 mM, and 250 mM) on anti-*Mtb* activity was evaluated via the resazurin microtiter assay (REMA). Stock solutions of the compounds to be evaluated and NaCl were prepared, and microdilutions were performed in 96-well plates to obtain final concentrations of 0.09 to 25.00 μg mL^–1^. One hundred microliters of *Mtb* inoculum were added to each well, and the plates were incubated at 37 °C for 7 days. On the seventh day, 30 μL of resazurin 0.01% (m/v) was added to each well. The fluorescence was measured 24 h later via a Cytation 3 (Biotek) plate reader.^12^

### 2.3 FEP Synergy with Established Anti-*Mtb* Antimicrobials

A potential synergistic effect of FEP with RIF, pretomanid, moxifloxacin, and delamanid on *Mtb* was evaluated using a checkerboard microdilution assay. Serial 2-fold dilutions of FEP were prepared in Middlebrook 7H9 medium supplemented with OADC, and 50 μL of each dilution was dispensed horizontally in the 96-well plate. Similarly, diluted concentrations of the reference antimicrobials were added vertically. Then, 100 μL of *Mtb* inoculum (10^5^ CFU/mL) was added to each well, except for column 12, which served as the sterility control. Plates were incubated at 37 °C for 7 days. The MIC was determined using the REMA method. After incubation, 30 μL of 0.01% (m/v) resazurin solution was added to each well, and fluorescence was recorded after 24 h using a Cytation 3 (Biotek) plate reader at excitation 530 nm and emission 590 nm.^13^

The Fractional Inhibitory Concentration index (FICI) of FEP in association with commercial drugs was calculated according to eq 1.

1 $$\mathrm{FICI}=\frac{\left(\mathrm{MIC}\;\mathrm{control}\;\mathrm{compound}\;\mathrm{in}\;\mathrm{association}\right)/}{\left(\mathrm{MIC}\;\mathrm{control}\;\mathrm{compound}\;\mathrm{alone}\right)}+\frac{\left(\mathrm{FEP}\;\mathrm{MIC}\;\mathrm{in association}\right)}{\left(\mathrm{FEP}\;\mathrm{MIC}\;\mathrm{alone}\right)}$$

According to standard interpretation criteria,^14^ FICI values are classified as synergistic (FICI ≤ 0.5), additive (FICI > 0.5 ≤ 1), neutral (FICI > 1 ≤ 2), or antagonistic (FICI > 2).

### 2.4 Field Emission Scanning Electron Microscopy (FE-SEM )

FE-SEM analysis was performed to evaluate the cell wall damage caused by FEP. Cultures of *Mtb* were exposed to 1× and 2× MIC_90_ FEP concentrations for 24, 48, and 72 h. INH and ethambutol were used as controls, along with an untreated group. After treatment, the samples were washed with PBS and fixed with Karnovsky solution (2% paraformaldehyde (v/v), 2% glutaraldehyde (v/v) and 0.1 M sodium cacodylate buffer pH 7.2). The samples were then stored at 4 °C overnight. The next day, the fixative was removed, and the samples were covered with cacodylate buffer. Afterward, cells were postfixed, for 30 min, in 1% osmium tetroxide (w/v) and 0.8% potassium ferricyanide (5 mM CaCl_2_ in 0.1 M sodium cacodylate buffer) for 30 min at 4 °C. The material was dehydrated in a graded acetone series (50–100%) for 10 min each. Then, the samples were critical-point-dried (Balzers, CPD 030, Germany) from liquid CO_2_ and gold-sputtered (SCD 500, LEICA-Germany). Images were obtained by JSM-7001F (Jeol Japan) scanning electron microscope (SEM).^15^

### 2.5 DNA Extraction and Whole-Genome Sequencing of FEP Spontaneously Resistant Mutants

To isolate mutants resistant to FEP, methods described by Gao et al.^16^ were used. Briefly, 7H11 agar plates with the test compounds at 1×, 2×, 4×, and 8× MIC_99_ concentrations were prepared. From an *Mtb* culture, 10^9^ CFU mL^–1^ were plated on 7H11 agar plates and incubated at 37 °C for 4 weeks. The resulting colonies were phenotypically characterized and plated on higher concentrations of FEP, and the process was repeated until no more colonies appeared. Colonies that grew on plates with the highest concentration were inoculated into liquid 7H9 medium with 1× MIC_90_ and incubated at 37 °C for 2 weeks. After washing with PBS, the mycobacteria were resuspended in freezing medium and stored at −80 °C.^17^ The mutants were thawed in 7H9 medium supplemented with OADC and grown under selective pressure equivalent to half the concentration used during their initial isolation. Once adequate growth was achieved in liquid medium, the culture was plated on 7H9 agar supplemented with the same selective pressure. Individual colonies were isolated from these plates for DNA extraction. The isolated colonies were resuspended in Eppendorf tubes with TE buffers and glass beads and shaken at maximum speed. The resulting supernatant was transferred to a new tube, to which 3 M sodium acetate and cold ethanol (96%, v/v) were added, and the mixture was shaken. After 1 h of incubation at room temperature, the samples were centrifuged, the supernatant was removed, 70% (v/v) ethanol was added, and the samples were incubated again under the same conditions. The supernatant was then removed, and the pellets were left to dry at room temperature. The dry pellet was resuspended in Milli-Q water and heated to 55 °C for 10 min with three cycles of shaking. Genomic DNA quantification was performed with a NanoDrop 2000 (Thermo Fisher Scientific Inc., Waltham, MA, USA). Genome sequencing was carried out at the Microbial Genome Sequencing Center (Pittsburgh, PA) via the HiSeq platform (Illumina Genome Analyzer, California, USA), with a minimum coverage of 100× and a read size of 300 base pairs paired end (150 × 150).^18^

### 2.6 Structural Modeling, Identification of Pharmacologically Active Pockets, and Molecular Docking

ChemDraw Professional 17.0 was used to draw the 2D structure of the compound and Chem3D to convert it into its 3D form. To simulate the temporal evolution of molecular structures and their behavior under different environmental conditions, molecular mechanics, energy, and geometric optimization treatments were varied within the Avogadro software.^19^ PockDrug was used to predict the receptor protein pockets capable of binding the compound, considering probabilities above 0.7 and a proximity of 5.5 Å.^20^ The crystalline structure of PonA1 (5CRF) was downloaded from the Protein Data Bank (https://www.rcsb.org). Binding energy calculations were performed to establish the affinity between target sites and ligand functional groups. Binding predictions between the optimized ligand structure and receptor pockets were made via AutoDock Vina, which is based on an empirical force field and the Lamarckian genetic algorithm.^14^ Discovery Studio Visualizer was used to analyze and generate 2D diagrams of the interactions of the compounds with the receptor protein target sites, and PyMOL was used to obtain 3D images of these interactions.^21^

### 2.7 Preparation of Nanostructured Lipid Systems (NLSs) and FEP-Loaded NLSs (NLS@FEP)

The NLS was composed of 10% oil phase (cholesterol), 10% surfactant (a mixture of soy phosphatidylcholine, sodium oleate, and Eumulgin HRE 40 [hydrogenated polyoxyl castor oil 40]; 3:6:8), and 80% aqueous phase (PBS pH 7.4). The mixture was sonicated via a rod sonicator (Q700 from QSonica, Newtown, CT, USA) operating at 700 W in discontinuous mode for 10 min at 30 s intervals every minute while being cooled in an ice bath. After sonication, the NLS was centrifuged at 11,180*g* for 15 min. FEP was first freeze-dried and then incorporated into the NLS (NLS@FEP) at 3 mg mL^–1^ through tip sonication for 3 min.^22^

### 2.8 High-Resolution Transmission Electron Microscopy (HR-TEM )

The size and morphology of the nanocarriers were characterized via a JEOL 1011 HR-TEM microscope (Tokyo, Japan) operated at 100 kV. The samples were prepared by placing a drop of the sample (5 μL) onto a 400-mesh copper grid, which was dried, and then negative staining was applied by using 5 μL of phosphotungstic acid (1%, m/v) for 10 min. The grids were air-dried at room temperature before analysis.

### 2.9 Mean Hydrodynamic Diameter, Polydispersity Index, and Zeta Potential Analysis

The mean hydrodynamic diameter and polydispersity index (PDI) were determined via dynamic light scattering (DLS), also known as quasielastic light scattering, as described by Silva et al.^23^ Zeta potential (ZP) analysis was performed by determining the electrophoretic mobility of the NLSs and NLS@FEP. The samples were diluted at a 1:10 (w/v) ratio in ultrapure water, and the parameters were analyzed via Zetasizer Nano NS equipment (Malvern Instruments, Malvern, UK).

### 2.10 Storage Stability

The storage stability of the nanosystems was assessed over 90 days at three different temperatures: 37.0 ± 0.5 °C, room temperature (25.0 ± 4 °C), and 5.0 ± 1.0 °C, as described by Araujo et al.^24^ The parameters include the hydrodynamic diameter, PDI, and ZP, which were measured via the same DLS equipment. The ZP (*n* = 3), mean hydrodynamic diameter and PDI (*n* = 10) were analyzed, and the mean values and standard deviations were calculated. Statistical significance was considered when *p* < 0.05, with a 95% confidence interval.

### 2.11 Stability Evaluation in Culture Media and Solutions with Different pH Values

The mean hydrodynamic diameter, PDI, and ZP were evaluated 24 h after diluting the NLS and NLS@FEP in 0.1 M HCl (pH 1.0) and PBS (pH 7.4), corresponding to the stomach and blood pH, respectively,^25^ to estimate the stability of the formulations under these conditions. The formulations were also evaluated in culture media used for cultivating *Mtb* (7H9) and Dulbecco’s modified Eagle’s medium (DMEM) with high glucose concentrations.^26^ Statistical significance was determined by two-way ANOVA, with *p* < 0.05 considered significant.

### 2.12 Iron Quantification via Inductively Coupled Plasma–Mass Spectrometry (ICP-MS )

The concentration of Fe was determined via ICP–MS at *m*/*z* 56 (^56^Fe^+^). To remove polyatomic spectral interferences, detection mode kinetic energy discrimination was employed. The calibration curve for Fe ranged from 0 to 500 μg L^–1^ when a multielemental stock solution (100 mg L^–1^) was used. All standards and samples were prepared in HNO_3_ (2%, v/v). A certified reference material, EnviroMAT Wastewater, Low (EU-L) (SPC Science, Canada), was also analyzed to ensure the quality of the measurements. A 100 μg L^–1^ solution of ^45^Sc and ^89^Y was used as an internal standard during the analyses. The limit of detection (LOD) for iron was 1.3 μg L^–1^.^27^

### 2.13 Evaluation of Entrapment Efficiency (EE) and Loading Capacity (LC)

The encapsulation efficiency (EE%) and drug loading capacity (LC%) of FEP were evaluated via ultracentrifugation with some modifications, as described by Laouini et al.^28^ First, 0.5 mL of NLS@FEP was centrifuged at 14,000 rpm at 4 °C for 30 min via a 5417R centrifuge (Eppendorf AG). Then, 250 μL of the supernatant was mixed with 250 μL of Triton X-100 (0.1%, m/v) to separate FEP from the other lipid components. The same dilution was performed for NLS@FEP, which had not undergone centrifugation. The samples were subjected to a 15 min ultrasonic bath, followed by centrifugation at 4,000 rpm for 5 min to sediment residual lipids. The total iron content of NLS@FEP was determined via ICP–MS, and the EE% and LC% were calculated via eqs 2 and 3, respectively.

2 $$\mathrm{EE}\%=\frac{\mathrm{Encapsulated}\;\mathrm{amount}\;\mathrm{of}\;\mathrm{drug}}{\mathrm{total}\;\mathrm{amount}\;\mathrm{of}\;\mathrm{drug}}\times 100$$

3 $$\mathrm{LC}\%=\frac{\mathrm{Encapsulated}\;\mathrm{amount}\;\mathrm{of}\;\mathrm{drug}}{\mathrm{total}\;\mathrm{nanosystem}\;\mathrm{content}}\times 100$$

### 2.14 *In Vitro* Release Kinetics

For the investigation of *in vitro* release, 20 mL of PBS buffer at pH 1.2 containing 1% polysorbate 80 was used at 37 °C with stirring at 150 rpm. Conditions were examined where the metal complex FEP was in its free form or in microemulsion form.^29^ The samples were placed in dialysis bags with a molecular weight cutoff of 12–14 kDa.^30^ Over a period of up to 24 h, aliquots were taken from the receptor medium at specific intervals (with replacement of the dissolution medium) and quantified via an inductively coupled plasma optical emission spectrometer (ICP-ES), Thermo Scientific model 6300, USA.

### 2.15 Infection and Treatment of BALB/C Mice

The study was conducted by adapting previous methods.^31–33^ Female BALB/c mice (6–8 weeks) were anesthetized with isoflurane and intranasally infected on day 0 with *Mtb* H37Rv at a total inoculum of 1.5 × 10^5^ CFU per animal. On day 14 postinfection, a sentinel cohort confirmed pulmonary infection; the remaining animals were randomized (*n* = 5 per group; CFU counts blinded to treatment) to receive vehicle (NLS), isoniazid (INH, 20 mg kg^–1^), free [Fe­(phen)_3_]^2+^ (FEP, 200 mg kg^–1^), or NLS@FEP (200 mg kg^–1^). From day 14 to day 42, dosing was delivered once daily by intratracheal instillation (40 μL per dose). Body weight and clinical condition (behavioral changes, coat appearance, and neurological signs) were monitored daily to assess animal health, and humane end points were predefined. On day 45, mice were euthanized; lungs were aseptically removed, homogenized, serially diluted, and plated on Middlebrook 7H11 + OADC. Plates were incubated at 37 °C for up to 50 days; the limit of detection (LOD) was 20 CFU per lung, and values below LOD were recorded as “not detected.” Data are shown as mean ± SD and analyzed by one-way ANOVA with Tukey’s test (α = 0.05). Animal procedures were approved by the institutional committee (protocol CEUA/FCF/CAr No. 17/2021).

## 3 Results and Discussion

### 3.1 Physiological Concentrations of NaCl Affect Antibacterial Sensitivity of*Mtb*H_37_Rv

Aiming to demonstrate the influence of NaCl concentration in antibacterial activities, the FEP was compared with RIF and INH under simulated different physiological environments: standard culture media (14.5 mM NaCl), human plasma (125 mM NaCl), and the intracellular environment of macrophages (250 mM NaCl) (Table 1).

**Table 1:** Effect of NaCl Concentration on MIC_90_ (μg mL^–1^) of FEP, RIF, and INH against *Mtb* H37Rv^a^

Compound	Not NaCl-Supplemented	14.5 mM NaCl (culture medium)	125 mM NaCl (plasma-like)	250 mM NaCl (macrophage-like)
FEP	3.92	1.42	0.36	0.89
RIF	0.098	0.098	0.098	0.19
INH	0.163	0.163	1.48	10.91

a MIC_90_: minimum inhibitory concentration that reduces growth by 90% relative to the untreated control. NaCl conditions emulate standard medium (14.5 mM), human plasma ionic strength (125 mM), and a macrophage-like environment (250 mM). Abbreviations: FEP, tris­(1,10-phenanthroline)­iron­(II); RIF, rifampicin; INH, isoniazid; NaCl, sodium chloride; Mtb, *Mycobacterium tuberculosis*.

Initially, in a medium without additional NaCl, FEP presented an MIC_90_ of 3.92 μg mL^–1^, indicating moderate antimicrobial activity against *Mtb* compared with RIF and INH (0.098 and 0.163 μg mL^–1^, respectively). As the NaCl concentration increased to 14.5 mM, the MIC_90_ of FEP decreased to 1.42 μg mL^–1^. Under conditions simulating human plasma (125 mM NaCl), the MIC_90_ of FEP decreased to 0.36 μg mL^–1^. When the intracellular environment of macrophages (250 mM NaCl) was simulated, the FEP MIC_90_ remained low (0.89 μg mL^–1^).

In contrast, the MIC_90_ of INH significantly increased under osmotic stress conditions, reaching 10.91 μg mL^–1^ in the simulated intracellular environment. RIF remained at 0.098 μg mL^–1^ from 0 to 125 mM NaCl and increased to 0.19 μg mL^–1^ at 250 mM NaCl.

The improvement of FEP at 14.5 and 125 mM NaCl, together with its still favorable activity at 250 mM, may be attributed to ion-induced changes in solubility or stability that enhance its antimicobacterial performance.^34^ This finding is particularly noteworthy because physiological NaCl concentrations often induce phenotypic tolerance, reducing the efficacy of many antibiotics; for instance, Larrouy-Maumus et al.^35^ reported that aminoglycosides lose potency under physiological salinity. In contrast, FEP exhibited enhanced activity, suggesting that it may circumvent these limitations.

By comparison, the marked decline in INH potency under osmotic stress is consistent with reports that NaCl-driven physiological adaptations in *Mtb* can compromise antibiotic effectiveness. Indeed, disruption of key proteins involved in osmoadaptation, such as PknD, has been shown to increase MICs of several antibiotics in *Mtb*.^36^ Additional evidence from Solcia et al.^37^ indicates that FEP activity is further improved under conditions that mimic granulomas, such as lower pH, and in the presence of serum proteins at physiological levels. Collectively, these observations highlight that FEP maintains its efficacy in host-mimicking environments and may even enhance it, an essential attribute given the intramacrophage niche of *Mtb*.

### 3.2 Synergy between FEP and Established Antimycobacterials

The potential synergistic interaction of FEP with clinically used antimycobacterials was evaluated against *Mtb* H37Rv (Table 2). For *Mtb*, an MIC_90_ of 3.92 μg mL^–1^ was observed for FEP alone. When combined with RIF, the MIC_90_ decreased to 0.07 μg mL^–1^, corresponding to a FICI of 0.27, which indicates synergy. The association with pretomanid yielded a FICI of 0.103, also within the synergistic range. The combination of FEP and delamanid resulted in a FICI of 0.69, consistent with an additive effect.

**Table 2:** FEP, RIF, Pretomanid, and Delamanid MIC_90_ Combined MIC_90_ and Fractional Inhibitory Concentration Indices (FICI) against *Mtb*, Expressed in μg mL^–1^

Pathogen	Drugs	MIC_90_	Combined MIC_90_	FICI	Result
*Mtb*	FEP	3.92	0.07	0.27	Synergistic
	RIF	0.004	0.001		
*Mtb*	FEP	3.92	0.07	0.103	Synergistic
	Pretomanid	0.35	0.03		
*Mtb*	FEP	3.92	0.07	0.69	Additive
	Delamanid	0.06	0.04		

The results demonstrate a strong synergistic interaction between FEP and RIF (FICI = 0.27), suggesting that this combination could potentiate standard TB treatment while allowing dose reduction and consequently minimizing RIF-associated toxicity. A similar synergistic effect was observed with pretomanid (FICI = 0.103), which reinforces the potential of FEP to improve therapeutic outcomes when used alongside newer antimycobacterial agents. In contrast, the association of FEP with delamanid resulted in an additive effect (FICI = 0.69). Although the degree of synergy was lower, the additive interaction still supports the possibility of reducing the required concentrations of both compounds, thereby improving tolerability and safety.

These findings are consistent with previous reports indicating that metal-based complexes can act on multiple cellular targets, thereby complementing the mechanisms of traditional antibiotics and enhancing their antimicrobial activity.^38,39^ The data highlight the versatility of FEP in combination therapies and point to its potential integration into multidrug regimens against *Mtb*.

### 3.3 Selection and Whole Genome Sequencing of Spontaneous FEP-Resistant Mutants

Whole-genome sequencing (WGS) was performed on the two spontaneous *Mtb* H37Rv mutants obtained after exposure to FEP, and the results were compared with the parental *Mtb* H37Rv strain. The analysis revealed eight single-nucleotide polymorphisms (SNPs) shared by both mutants and absent from the wild-type genome. After filtering out highly polymorphic genes, two relevant mutations remained, located in ponA1 and pks1. Phenotypically, the FEP-resistant mutants showed markedly slow growth. In 7H9 liquid medium supplemented with 10% OADC under selective pressure equivalent to the FEP MIC_90_, cultures required about 50 days to reach a turbidity equivalent to McFarland 1. On solid medium, colonies appeared only after ∼60 days under the same selective conditions.

The PonA1 gene encodes a bifunctional penicillin-binding protein (PonA1) that catalyzes peptidoglycan transglycosylation and transpeptidation, acting as a regulator of polar growth in mycobacteria.^40^ The pks1 gene encodes a polyketide synthase associated with biofilm formation. Although PBPs are not classical targets in *Mtb*—since endogenous β-lactamases degrade most β-lactams—recent studies have demonstrated that β-lactam antibiotics can display activity against both susceptible and resistant *Mtb* strains.^41^

Phosphorylation of PonA1 is essential to control the rate of polar growth, and altered expression levels lead to abnormal morphologies, highlighting its critical role in maintaining proper cell wall synthesis.^40^ Farhat et al.^42^ further showed that PonA1 influences tolerance to antibiotics such as RIF, underscoring its connection to cell-wall-targeting therapies. Disruption of PonA1 enzymatic activity or phosphorylation state also impacts bacterial susceptibility to other inhibitors of peptidoglycan biosynthesis, including teicoplanin.

The slow-growth phenotype of the FEP-resistant mutants resembles the behavior reported in *Mycobacterium smegmatis* with disruption of pbp1, the ortholog of ponA1 in *Mtb*.^43^ Altogether, the identification of mutations in PonA1 and pks1, combined with the observed growth defect, suggests that FEP may act by interfering with cell wall synthesis, possibly through PonA1 inhibition.

### 3.4 *In Silico* Results

The 2D-modeled drug structure was converted to 3D in Chem3D, and the ligand was relaxed/optimized in Avogadro (Figure S1). PocketDrugg predicted 35 druggable pockets in PonA1; for this study, nine pockets with >70% druggability were prioritized (Table S1). Models were selected based on FEP–PonA1 affinity, interaction counts, and bond types (Table S2).

Docking (AutoDock Vina) predicted favorable FEP binding across several PonA1 pockets, with calculated energies from −10.7 to −8.2 kcal mol^–1^. For context, Mundhe et al.^44^ reported −5.1 kcal mol^–1^ for INH-InhA. Pocket 10 showed the most favorable score and lies in the region where penicillin V binds PonA1 in the cocrystal PDB 5CXW.^45^ Interaction analysis (Discovery Studio Visualizer) showed that the nine prioritized pockets contact the ligand, with hydrophobic contacts predominating (Figure 1). Pockets 4, 6, 9, and 24 exhibited electrostatic interactions. Pockets 3 and 7 formed nonclassical hydrogen-bond interactions involving aromatic rings. 3D images were generated in PyMOL.

**Figure 1:**
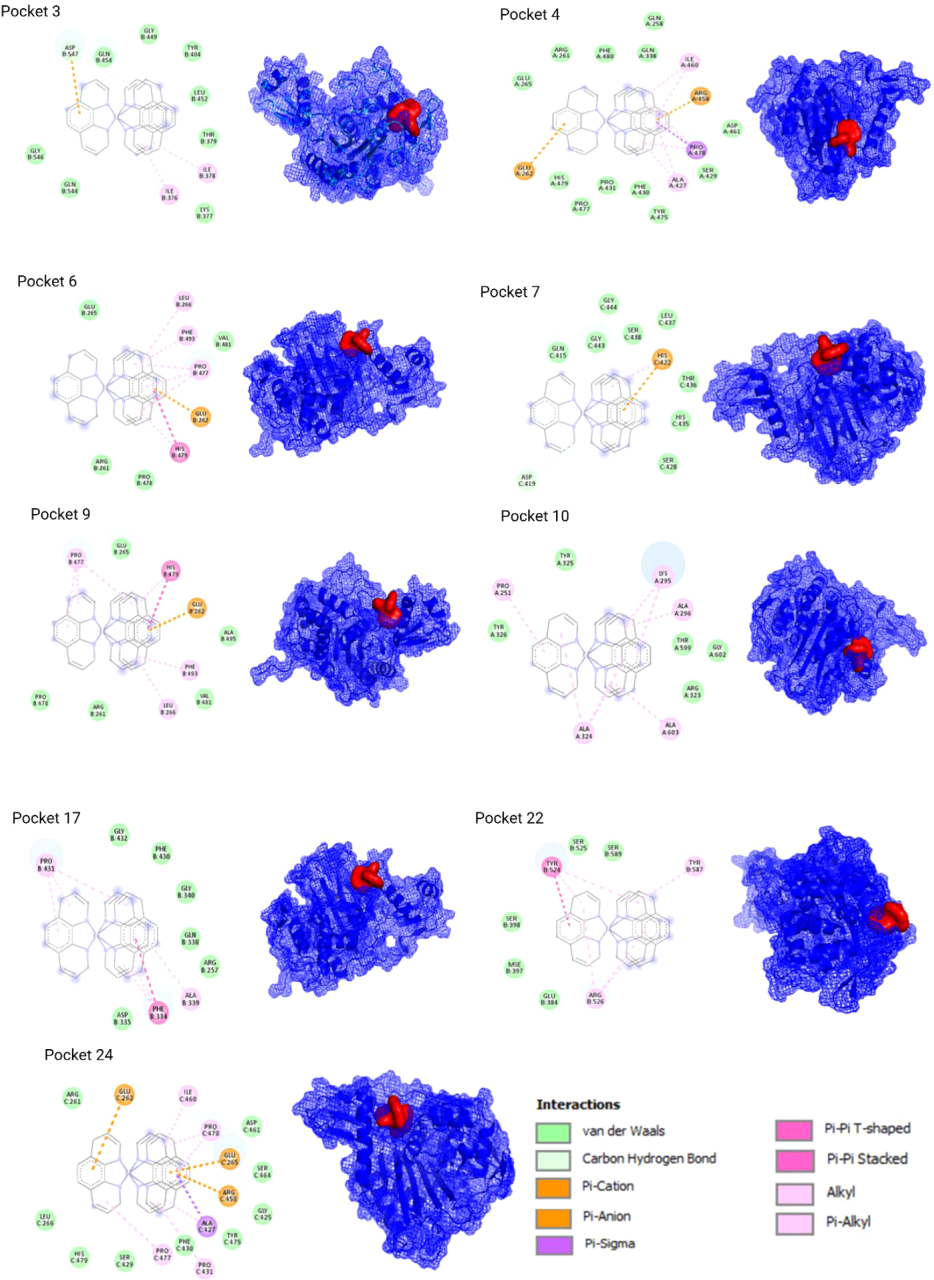
Representative 2D and 3D docking models of FEP interacting with selected PonA1 pockets predicted by PockDrug (>70% druggability). Interaction types are color-coded (hydrophobic, electrostatic, hydrogen bonds, π-interactions).

The docking scores indicate predicted FEP–PonA1 affinities that are more favorable (in absolute value) than the INH–InhA reference,^44^ and the best-scoring pose is located near the penicillin V site in 5CXW.^45^ The verified interaction profile—hydrophobic contacts together with electrostatic contributions (pockets 4, 6, 9, 24) and nonclassical hydrogen bonds (pockets 3, 7)—suggests engagement features that extend beyond hydrophobic complementarity. Considering these *in silico* observations with the ponA1 mutations (Section 3.3) and the FE-SEM morphology (aberrant poles and altered cell length), a plausible mechanistic scenario emerges in which FEP may interfere with processes linked to transglycosylation/transpeptidation and cell-wall synthesis by engaging PonA1. This remains a hypothesis. Accordingly, docking scores should be interpreted with caution: they prioritize plausible binding modes but may overestimate affinity in the absence of biochemical assays or PonA1 mutant analyses. Further orthogonal validation, such as molecular dynamics-based free-energy estimates, mutagenesis of pocket residues, and biochemical assays of PonA1 activity, will be required to confirm these computational predictions.

### 3.5 FEP Impacts on *Mtb* Cell Wall Integrity

To further explore the possible mechanism suggested by genomic and *in silico* analyses, we evaluated the morphology of *Mtb* exposed to FEP through FE-SEM and compared the findings with untreated controls and with cells exposed to reference drugs known to interfere with cell-wall synthesis. In the untreated control (Figure 2A), the bacilli displayed the expected rod-like morphology with smooth surfaces and intact structures, which served as a baseline. After treatment with ethambutol at 1 × MIC_90_ (Figure 2B), the bacilli showed surface irregularities and cellular debris, indicating a loss of envelope integrity. In cells treated with isoniazid at 1 × MIC_90_ (Figure 2C), amorphous structures and signs of lysis were observed, accompanied by an accumulation of disorganized debris. In contrast, bacilli exposed to FEP at 1 × MIC_90_ (Figure 2D) displayed roughened surfaces, elongated and flattened forms, and an accumulation of nonviable material, with the additional observation that no dividing cells were detected.

**Figure 2:**
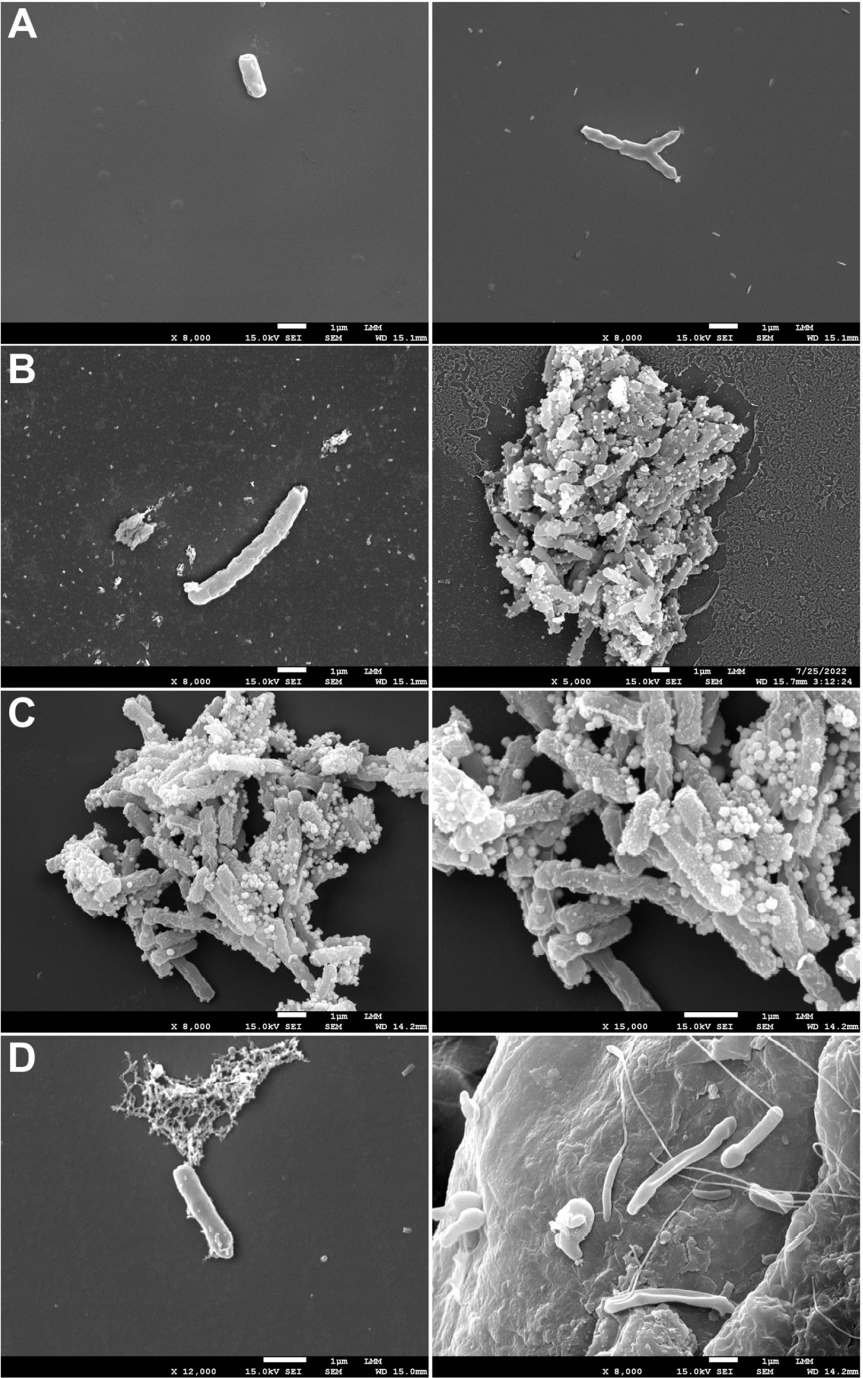
(A) Untreated bacilli displaying normal rod-like morphology with intact surfaces. (B) Cells exposed to EMB at 1× MIC_90_ showing surface alterations and cellular debris consistent with impaired envelope structure. (C) Cells treated with INH at 1× MIC_90_ presenting amorphous remnants and lytic features indicative of collapsed bacillary architecture. (D) Cells treated with FEP at 1× MIC_90_ exhibiting roughened surfaces, elongated and flattened bacilli, accumulation of nonviable material, and absence of dividing cells. Scale bars are indicated in each panel.

The FE-SEM analysis revealed characteristic alterations associated with established antimicrobials. Ethambutol produced surface damage and cellular debris, consistent with inhibition of arabinogalactan biosynthesis, whereas isoniazid caused extensive lysis and collapse of bacillary structures, reflecting its known inhibition of mycolic acid synthesis.^46^ When evaluating the FEP-treated bacilli, the roughened surfaces, abnormal elongation and flattening, and absence of dividing cells suggest interference with pathways essential for maintaining cell-wall integrity. These morphological effects resonate with previous reports that linked PonA1 activity to envelope structure and polar growth in *Mtb*,^40^ and they complement the genomic evidence of ponA1 mutations (Section 3.3) as well as the *in silico* results indicating strong FEP–PonA1 affinity (Section 3.4).

### 3.6 Synthesis and Characterization of the NLSs and NLS@FEP

The NLSs were prepared via an oil phase and surfactant mixture following a previously described protocol,^22^ with subsequent tip sonication producing a pearly coloration and opaque liquid as shown in Figure 3A. FEP loading was performed by adding FEP at 3 mg mL^–1^ to NLS followed by tip sonication for 3 min resulting in a strong reddish coloration, typical of the iron complex, and without any apparent change in viscosity, as shown in Figure 3B.

**Figure 3:**
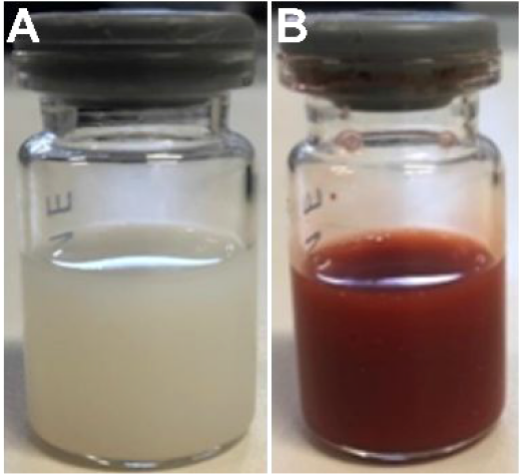
Representative images of the fabricated nanosuspensions. (A) NLS, showing a pearly and opaque appearance after sonication, characteristic of the lipid dispersion. (B) NLS@FEP, exhibiting an intense reddish coloration, typical of the FEP, with no apparent changes in suspension viscosity.

To confirm the fabrication of the nanosystems as well as FEP loading, mean hydrodynamic diameter, polydispersity indices and ZP were determined (Figure 4). Figure 4A shows an average hydrodynamic diameter of 140.5 ± 3.4 nm and 162.97 ± 0.35 nm, for NLS and NLS@FEP, respectively, with good stability in ultrapure water with PDIs of up to 0.159. The ZP results (Figure 4B) show that the negative surface charge of the NLS slightly decreased after FEP loading (−38.5 ± 2.8 and −34.7 ± 2.2 mV, respectively), suggesting that FEP was successfully loaded, and is not confined to/present on the outer NLS surface. HR-TEM images (Figure 5) revealed that both NLS and NLS@FEP are spherical with uniform diameter distributions, consistent with the DLS measurements.

**Figure 4:**
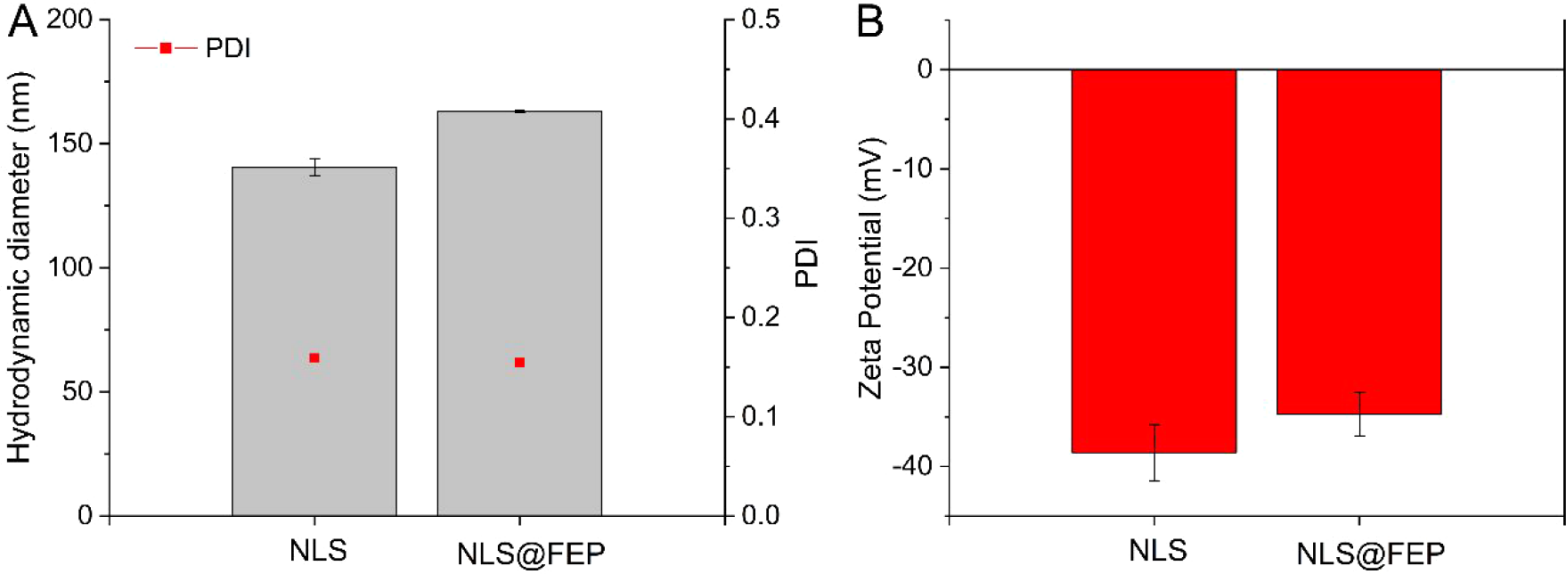
Physicochemical characterization of the nanosystems. (A) Mean hydrodynamic diameter and PDI values of NLS and NLS@FEP, showing a slight size increase after FEP incorporation while maintaining low PDI. (B) ZP measurements indicating a moderately negative surface charge for both formulations, with a minor reduction upon FEP loading (in ultrapure water, *n* = 3).

**Figure 5:**
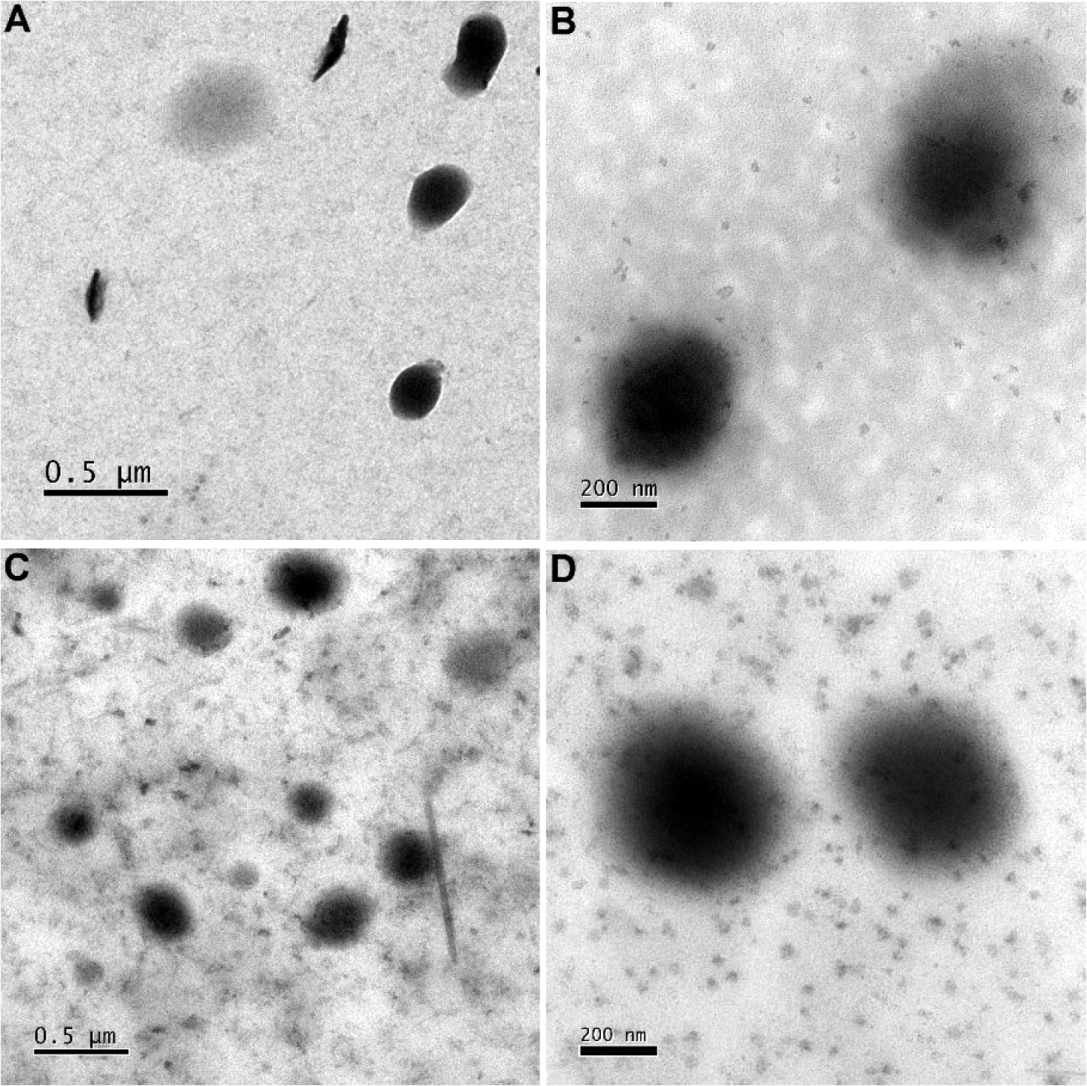
HR-TEM images of (A, B) NLSs at 5,000× and 10,000× magnification, respectively, and (C, D) NLS@FEP at 5,000× and 10,000× magnification, respectively.

After NLS@FEP fabrication, the LEP EE and LC were calculated, with values of 57.72 ± 6.00% and 0.35 ± 0.06%, respectively. These values agree with the literature considering that the interaction between the hydrophilic FEP and the lipid within the nanostructured system is crucial for effective drug encapsulation and loading.^26^ Cavalcanti et al.^47^ developed zidovudine-loaded NLSs and reported EE and LC values of 44.00 and 0.31%, respectively. Similarly, Gambhire et al.^48^ reported EE values ranging from 51.33 to 71.80% when optimizing dithranol-loaded lipid nanoparticle formulations.

### 3.7 Physical Stability during Storage of NLS and NLS@FEP

The physical stability of the NLS and NLS@FEP at different temperatures over time was determined (Figure 6). At 5 °C, the formulations showed no significant changes in mean hydrodynamic diameter over 90 days compared to the initial measurements, with 145.53 ± 0.90 nm and 156.90 ± 4.49 nm for NLS and NLS@FEP, respectively. In addition, the PDI remained stable at 0.15 ± 0.01 for both formulations. ZP values at 5 °C did were not significantly different up to 90 days, fluctuating between −38.33 ± 2.18 mV and −44.00 ± 2.78 mV and between −33.80 ± 1.47 mV and −37.60 ± 1.61 mV for NLS and SLN@FEP, respectively (Figure 6A).

**Figure 6:**
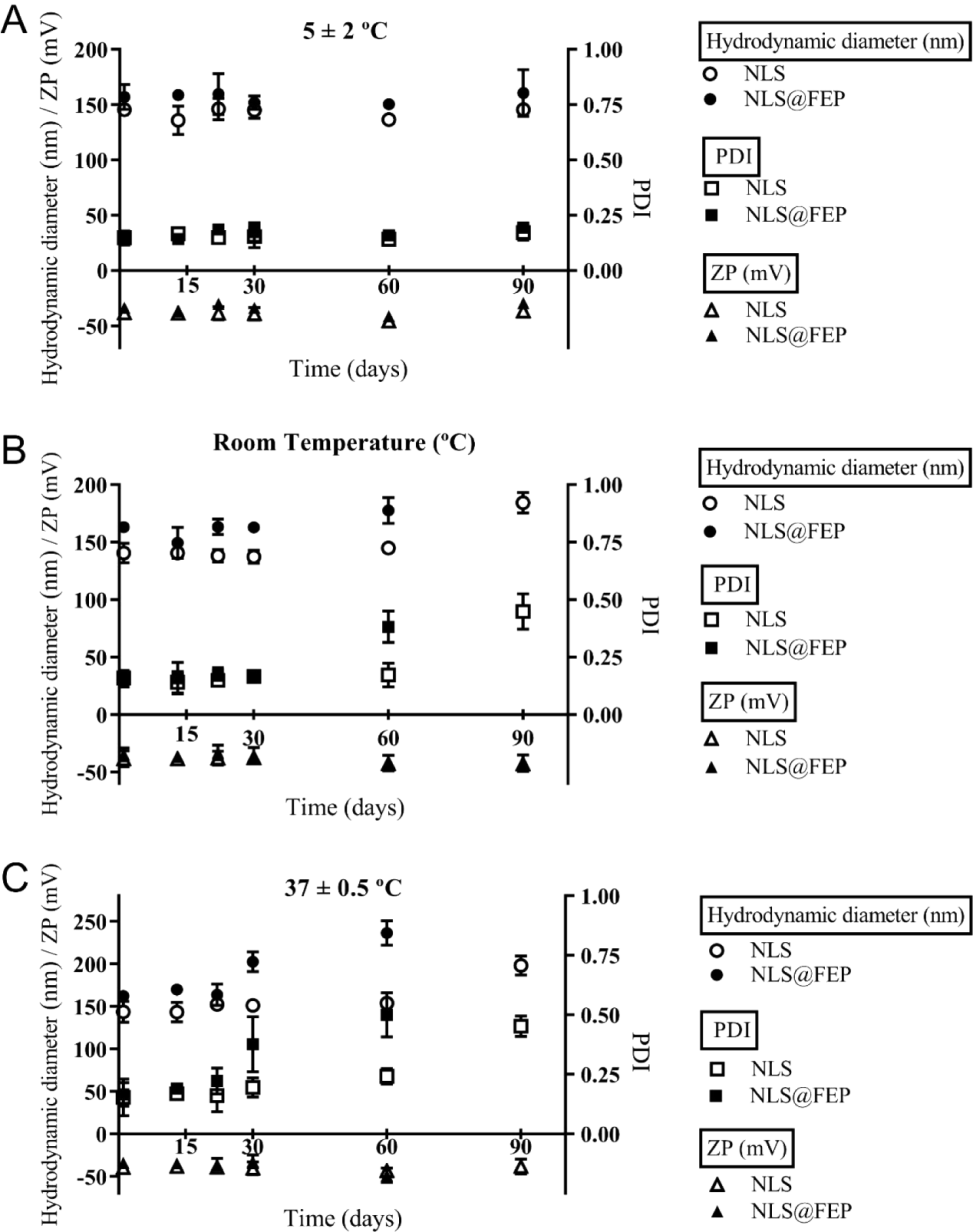
Stability profiles of NLS and NLS@FEP at different storage conditions. Mean hydrodynamic diameter, polydispersity index (PDI), and zeta potential (ZP) were monitored for 90 days at (A) 5 °C, (B) room temperature, and (C) 37 °C. Data are presented as mean values with 95% confidence intervals (*n* = 3).

At room temperature, NLS did not display significant changes in average diameter and PDI up to day 60. However, an increase in average diameter from 144.9 ± 1.0 nm to 184.4 ± 3.6 nm and in PDI from 0.17 ± 0.02 to 0.45 ± 0.03 was observed on day 90. There was a significant increase in NLS@FEP average diameter and PDI from day 60, rising from 162.97 ± 0.35 nm and 0.16 ± 0.02 to 177.43 ± 4.47 nm and 0.38 ± 0.03, respectively, at day 90. In addition, ZP values varied from −38.57 ± 2.82 mV to −43.00 ± 1.64 mV and from −34.70 ± 2.26 mV to −46.10 ± 1.51 mV for NLS and NLS@FEP, respectively (Figure 6B).

At 37 °C, NLS remained stable (*p* < 0.05) in terms of average diameter and PDI up to day 30. From day 60 to day 90, there was a slight increase in average diameter and PDI rising from 143.70 ± 4.91 nm and 0.15 ± 0.03 to 153.80 ± 1.29 nm and 0.24 ± 0.01, respectively. In contrast, there was a significant increase (*p* < 0.05) in the average diameter and PDI of NLS@FEP at days 30 (162.40 ± 1.83 nm and 0.17 ± 0.02, respectively) and 90 (236.30 ± 5.82 nm and 0.50 ± 0.04, respectively). Both formulations had significant decreases in ZP (*p* < 0.05) by day 60, varying from −41.30 ± 2.00 mV to −46.30 ± 1.85 mV and from −34.00 ± 2.00 mV to −48.63 ± 3.45 mV for NLS and NLS@FEP, respectively (Figure 6C). The NLS@FEP stored at room temperature and 37 °C were not analyzed on day 90 due to the formation of a surface film caused by water evaporation—a phenomenon associated with capillary forces from dehydration—as previously observed by Müller et al.^49^ The increases in average hydrodynamic diameter and PDI, along with the decrease in ZP, suggest the formation of aggregates through particle coalescence, an effect that intensifies with higher temperatures. Despite these changes, both nanosystems demonstrated notable uniformity and stability when stored at 5 °C for up to 90 days.

### 3.8 Stability in Physiological Conditions of NLS and NLS@FEP

After 24 h in PBS pH 7.4, both NLS and NLS@FEP exhibited increases in average diameter of 4.03 and 3.40 nm, respectively. ZP values varied by ±7.03 mV and ±8.40 mV for NLS@FEP compared to their counterparts diluted in distilled water. In 0.1 M HCl pH 1.0, minimal and nonsignificant increases in average diameter were observed (0.4 and 0.16 nm for NLS and NLS@FEP, respectively). ZP values varied by ±4.76 mV and ±5.97 mV for NLS and NLS@FEP, respectively. PDI values remained stable under all tested conditions.

The slight increases in hydrodynamic diameter observed in PBS can be attributed to the presence of salts that enhance the solvation layer considered in DLS measurements.^50^ Variations in ZP values in both PBS and HCl suggest ion adsorption from the solutions, which interferes with accurate ZP determination.^51^ Despite these fluctuations, the stability of PDI values indicates that no aggregation occurred, confirming that both nanosystems retained their structural integrity under physiological neutral and acidic pH conditions (Figure 7).

**Figure 7:**
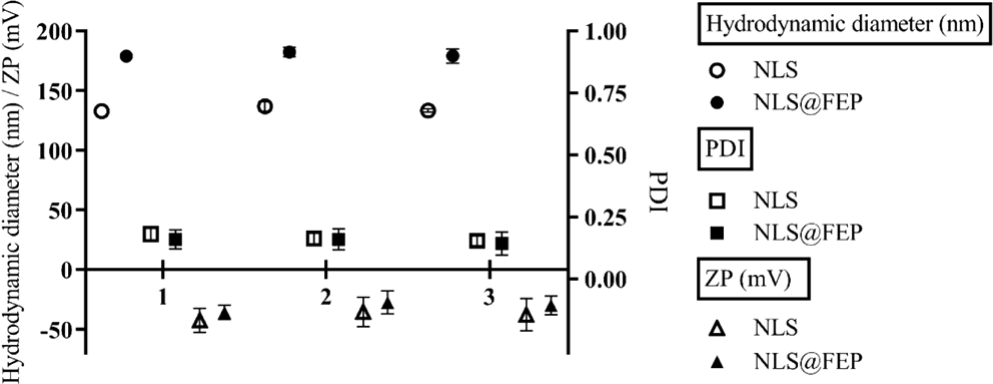
Hydrodynamic diameter, polydispersity index (PDI), and zeta potential (ZP) of NLS and NLS@FEP after 24 h of dilution in different media: (1) distilled water, (2) PBS pH 7.4, and (3) 0.1 M HCl (*n* = 3).

Figure 8 shows an increase in the average diameter of approximately 15 nm for NLS and 11 nm for NLS@FEP when diluted in high-glucose DMEM and 7H9 culture media. PDI values remained consistent under both conditions, indicating the absence of aggregate formation. Significant variations in ZP were also detected: NLS shifted from −49.40 mV to −13.60 mV and −7.50 mV in 7H9 and DMEM, respectively, while NLS@FEP shifted from −38.10 mV to −10.90 mV and −7.80 mV under the same conditions.

**Figure 8:**
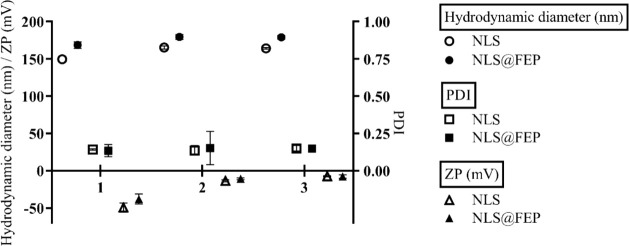
Hydrodynamic diameter, polydispersity index (PDI), and zeta potential (ZP) of NLS and NLS@FEP after 24 h of dilution in different media: (1) distilled water, (2) 7H9 medium, and (3) high-glucose DMEM (*n* = 3).

The observed increase in hydrodynamic diameter is attributed to ion adsorption from the media onto the nanosystem surface, leading to the formation of larger solvation layers.^50^ The stability of PDI values indicates that no aggregation occurred, consistent with previous findings by Silva et al.,^52^ who reported that solid lipid nanoparticles did not form aggregates in DMEM. The pronounced changes in ZP also reflect ion adsorption from the media.^51^ Importantly, both nanosystems maintained a negative surface charge in all tested conditions, which is beneficial since negatively charged particles exhibit lower serum protein adsorption, thereby enhancing their circulation time *in vivo*.^53^

### 3.9 *In Vitro* FEP and NLS@FEP Release Profile

The *in vitro* release kinetics assays were conducted under sink conditions, ensuring that the dissolution medium could dissolve at least three times the FEP amount used. Figure 9 shows the release profile of free FEP and NLS@FEP at 37 °C in PBS, pH 1.2, simulating the fasting stomach environment. Free FEP exhibited rapid release, reaching approximately 40% cumulative release within the first hour, remaining stable up to 20 h, followed by a slight decrease. In contrast, NLS@FEP displayed a slower and lower release, reaching only about 10% after 12 h.

**Figure 9:**
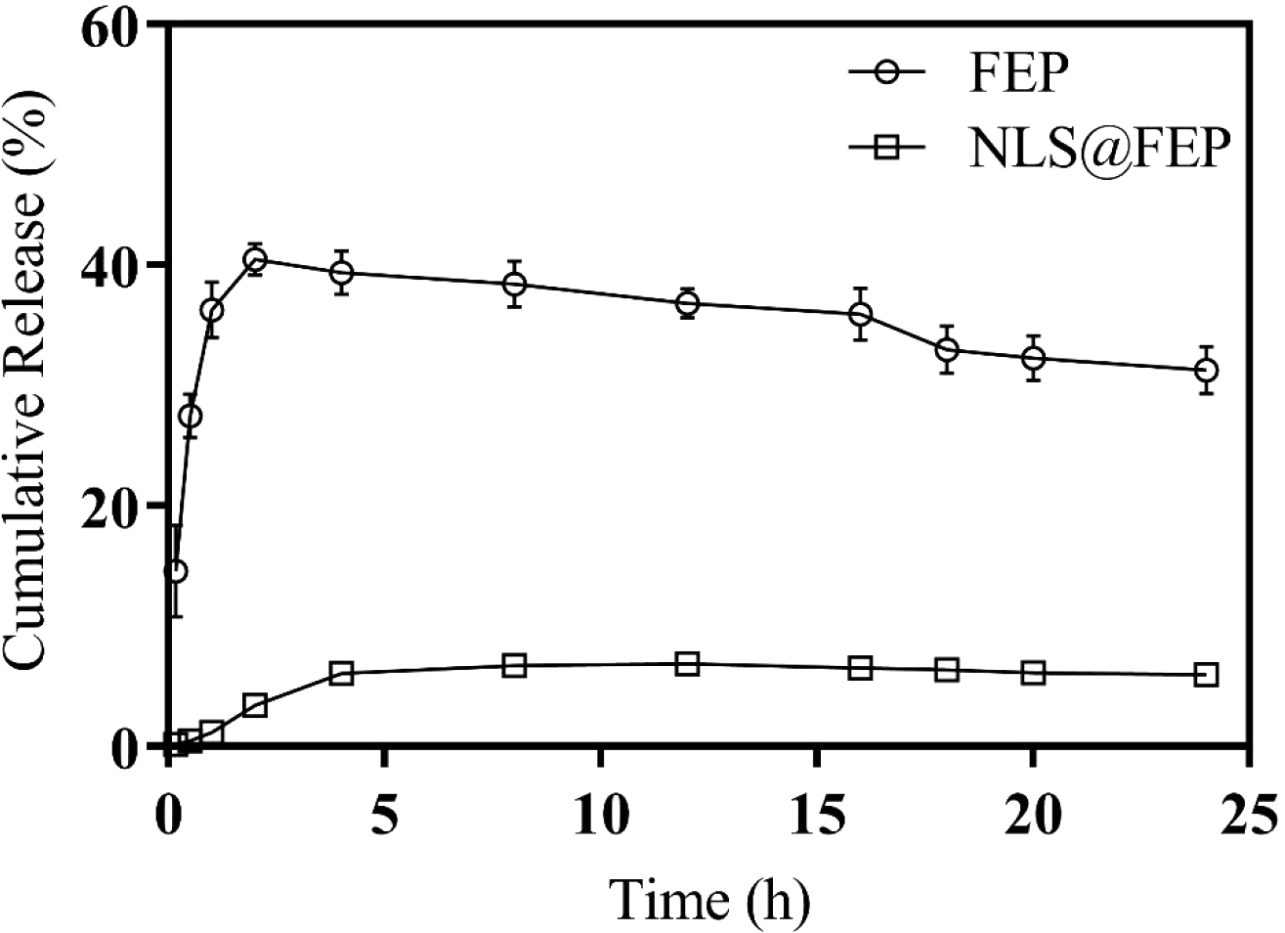
*In vitro* cumulative release profiles of free FEP and NLS@FEP in PBS (pH 1.2) at 37 °C over 24 h. Free FEP showed rapid release reaching ∼40% within 1 h, whereas NLS@FEP exhibited a slower and sustained release not exceeding ∼10%.

The slower release of NLS@FEP observed *in vitro* contrasts with *in vivo* data reported by Solcia et al.,^18^ where detectable levels of NLS@FEP were present in the bloodstream 6 h postadministration at concentrations sufficient to inhibit mycobacterial growth. This discrepancy may be attributed to the acidic conditions (pH 1.2) used in the assay, as pH-dependent release of lipid-based nanoparticles has been previously described Kalhapure et al.,^54^ The therapeutic relevance of these release patterns depends on the clinical context: in acute infections, immediate release is preferred to achieve rapid bactericidal action, while in chronic infections such as TB, a slower, sustained release is more beneficial for maintaining consistent therapeutic levels over extended periods.^55^

TB can manifest in various forms, from an initial acute infection to a latent or active chronic infection. In this context, controlled drug release could help minimize the risk of developing antibiotic resistance by avoiding fluctuations in plasma concentrations that could favor the survival of resistant bacteria.^56^ Prolonged exposure to subtherapeutic concentrations contributes to the emergence of resistant strains, so a formulation that maintains optimal concentrations is essential for therapeutic success. Moreover, for localized infections, a slower release may be beneficial for maintaining high antibiotic concentrations at the target site of infection, improving the local efficacy of antibiotics and reducing unwanted systemic effects. The NLS@FEP can offer advantages by protecting the drug from degradation, allowing controlled and targeted release.^57^

The frequency of administration and adherence to treatment are critical factors in TB management. Prolonged and complex therapeutic regimens can lead to patient noncompliance, which compromises treatment efficacy and promotes the development of resistance.^58^ A slow-release formulation that allows for reduced administration frequency could significantly improve adherence, increasing therapeutic success rates.

### 3.10 FEP and NLS@FEP Efficacy in *In Vivo* Assay

Throughout the 4-week regimen (days 14–42 postinfection (p.i.)), the animals maintained the expected weight gain and showed no relevant behavioral changes, consistent with good tolerability under the dosing conditions (Figure 10A). A spontaneous death was recorded in the vehicle group during the first week; no losses occurred in the treated groups. At the end point (day 45 p.i.), lungs were homogenized and colonies were enumerated after extended incubation; CFU counts were performed approximately 50 days after the experiment to maximize detection of residual growth.

**Figure 10:**
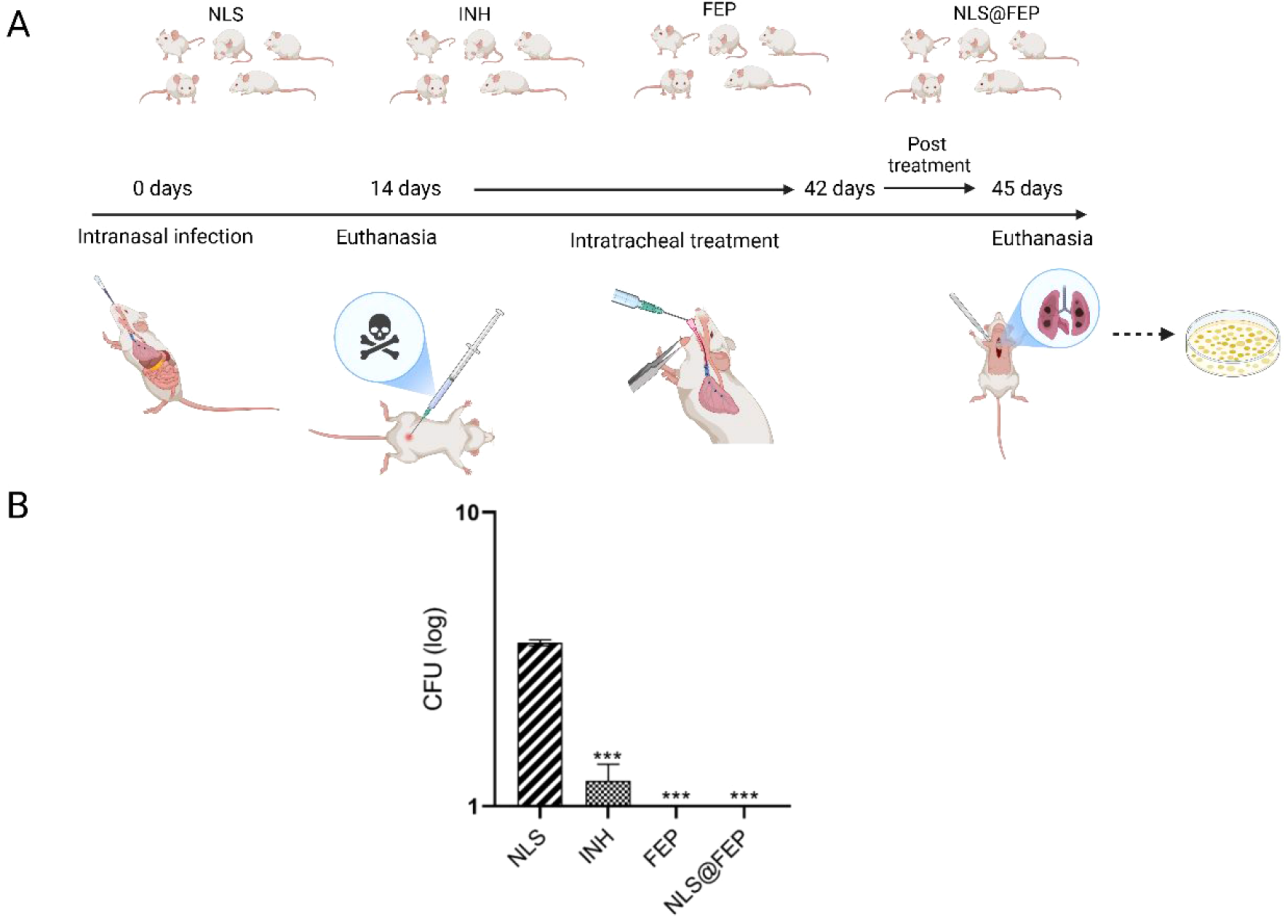
*In vivo* evaluation of free FEP and NLS@FEP in BALB/c mice infected with *Mtb* H37Rv. (A) Experimental scheme showing infection, treatment initiation on day 14, and end point lung CFU determination. (B) Lung CFU counts after 4 weeks of intranasal therapy with NLS, INH, free FEP, or NLS@FEP. Bars represent mean ± SD (*n* = 5 per group); ***p < 0.05 compared with the NLS control.

Under these conditions, the vehicle group (NLS) showed an approximate ∼1.5 log_10_ reduction from baseline, compatible with innate host responses. Compared with vehicle, INH (20 mg kg^–1^) produced an additional ∼2 log_10_ reduction, confirming its expected efficacy. In contrast, lungs from animals treated with free FEP (200 mg kg^–1^) or NLS@FEP (200 mg kg^–1^) showed no detectable growth after extended incubation, indicating sterilization within the detection limits of the assay. Data are presented as mean ± SD (*n* = 5), and statistical analysis shown in the figure indicates significant differences versus NLS (*p < 0.05) (Figure 10B).

No difference was resolved between FEP and NLS@FEP in this experiment (same visual conclusion in Figure 10B), suggesting that the intrinsic potency of FEP was sufficient to reduce bacterial burdens below the detection threshold in this acute model; any additional advantage attributable to the nanoformulation will require designs that disentangle release and pharmacokinetic effects.

The absence of detectable CFU in lungs from animals treated with FEP or NLS@FEP, together with the partial reductions observed in the control and INH groups under the same conditions, suggests a higher efficacy of FEP-based regimens in this model. This outcome is consistent with the mechanistic scenario proposed across Sections 3.3–3.5, where genomic, docking and FE-SEM data converge toward PonA1 as a plausible node linked to cell-wall processes; prior literature indicates that perturbation of PonA1 compromises peptidoglycan biogenesis and envelope integrity in *Mtb*, a context that can lead to loss of viability.^40^ It is important to note, however, that *in silico* assays are probabilistic in nature and provide only theoretical predictions of interaction affinity and geometry, which do not ensure biological activity *in vivo*.

No difference was detected between free FEP and NLS@FEP in this experiment; nevertheless, nanostructured lipid systems have been reported to enhance bioavailability and tissue distribution and to facilitate targeting, attributes that could translate into benefits under dosing schemes optimized for release kinetics and pharmacokinetics.^59^ Future studies designed to resolve exposure–response relationships and to quantify drug levels in lung compartments would help determine whether NLS-based delivery provides an advantage over the free compound in this indication.

## 4 Conclusion

This study highlights the remarkable antimycobacterial potential of FEP, which exhibited strong activity against Mtb with an MIC90 value of 3.92 μg mL^–1^. The synergistic interactions of FEP with RIF and pretomanid, leading to reduced MICs and favorable FICI indices, underscore its capacity to optimize existing therapeutic regimens by enabling lower dosages and reducing the risk of adverse effects associated with high antibiotic concentrations. FE-SEM studies and genomic analyses suggest that FEP interferes with peptidoglycan synthesis, a critical component of the mycobacterial cell wall, likely through interaction with PonA1, as supported by molecular modeling; however, definitive confirmation will require biochemical and genetic validation. The complete elimination of pulmonary infection in a murine model of *Mtb* H37Rv infection, achieved with both free FEP and FEP-loaded nanostructured lipid systems (NLS@FEP), demonstrated superior efficacy compared with isoniazid. The NLS@FEP formulation enhances the stability and bioavailability of the complex while providing additional benefits such as controlled release and reduced toxicity, reinforcing its potential for long-term clinical applications. Despite these promising findings, certain limitations must be acknowledged. Docking analyses may overestimate binding affinities, and the proposed interaction with PonA1 remains hypothetical in the absence of direct functional evidence. Moreover, although *in vivo* efficacy was demonstrated in a murine model, the potential toxicity of FEP and its long-term safety profile remain unknown. Future work should therefore prioritize systematic toxicity assessments, including both acute and chronic studies, together with pharmacokinetic characterization. Expanding efficacy testing to advanced TB models, including those simulating drug-resistant or latent infections, and validating PonA1 as a therapeutic target through biochemical and genetic approaches will be critical to strengthen the translational potential of this strategy.

## Supplementary Material



## Notes

In silico results, 2D structure of FEP, characteristics of FEP-druggable pockets in the PonA1 receptor, Docking affinity of the FEP (DOCX)
