# Supplementary material for: [Fe(phen) 3 ] 2+ and [Fe(phen) 3 ] 2+ -Loaded Nanostructured Lipid System: In Silico, In Vitro, and In Vivo Efficacy against Mycobacterium tuberculosis
Source: ACS Omega. 2025 Nov 27;10(48):59145–58. doi: 10.1021/acsomega.5c08350 (PMC12771237; doi:10.1021/acsomega.5c08350)
Supplement: Supplementary file 1 [file ao5c08350_si_001.docx]

**[Fe(phen)_3_]^2+^ and [Fe(phen)_3_]^2+^-Loaded Nanostructured Lipid System** ***In silico,* *In vitro* and *In vivo* Efficacy Against *Mycobacterium tuberculosis***

Fernanda Manaia Demarqui^1†^, Christian Shleider Carnero Canales^2†^, Rachel Temperani Amaral Machado^1^, Rafael Miguel Sábio^1^, Ingrid Gracielle M. Silva^3^, Karine B. Barros-Cordeiro^3^, Sônia N. Báo^3^, Masanori Asai^4^, Sandra M. Newton^4^, Paul R. Langford^4^ and Fernando Rogério Pavan^1*^.

^1^Tuberculosis Research Laboratory, School of Pharmaceutical Sciences, São Paulo State University – UNESP, Araraquara, São Paulo, Brazil

^2^Vicerrectorado de Investigación, Universidad Autónoma del Perú, Lima, Perú

^3^Microscopy and Microanalysis Laboratory, Department of Cell Biology, Institute of Biological Sciences, University of Brasília. 70365-070 Brasília - DF, Brazil

^4^Paediatric Infectious Diseases, Department of Infectious Disease - Faculty of Medicine, Imperial College, London, England

*** Corresponding author.** E-mail address: fernando.pavan@unesp.br (F. R. Pavan)

†These authors contributed equally to this work

1. ***In silico* results**


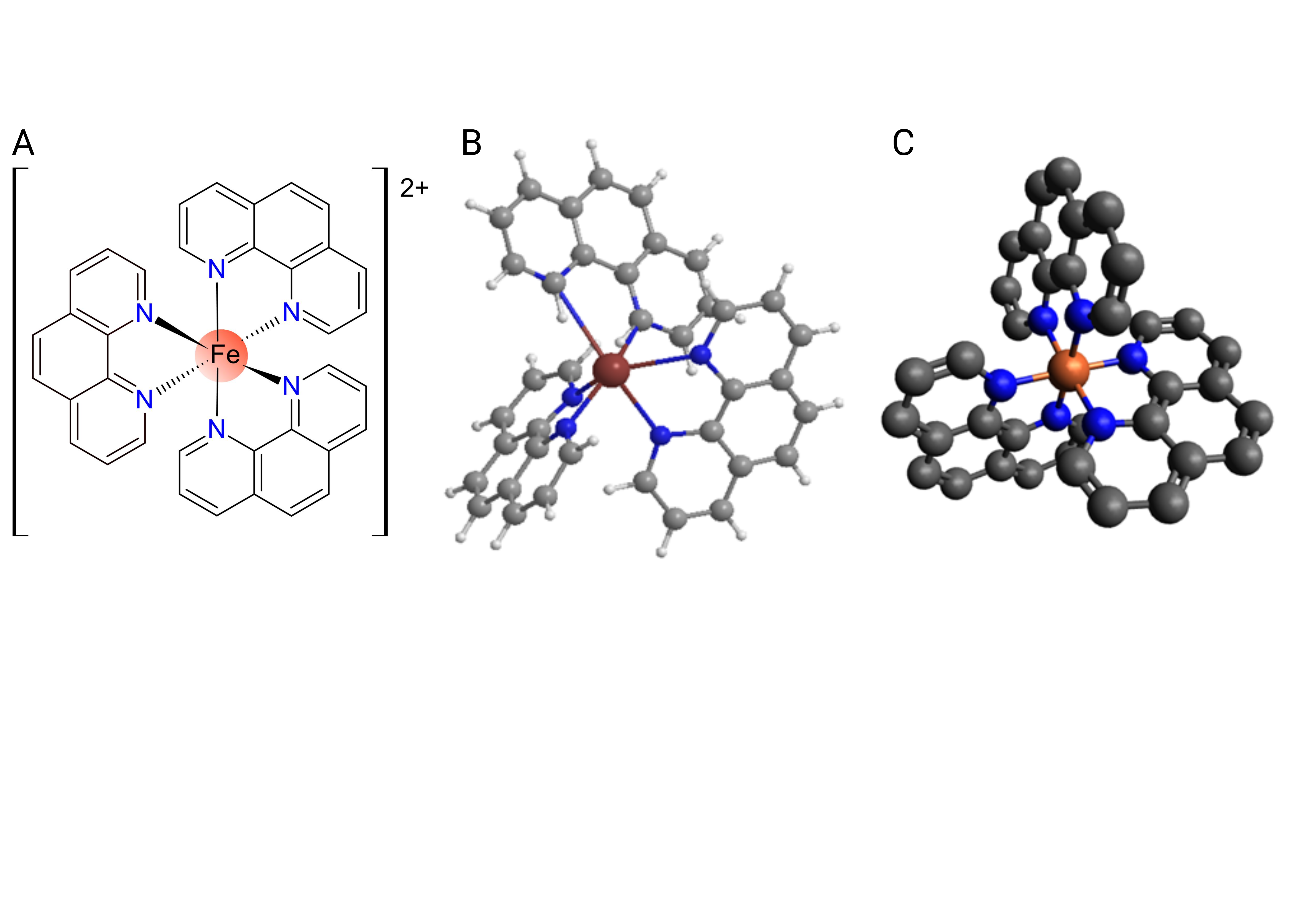


**Figure S1.** **A**) 2D structure of FEP, **B**) 3D structure generated with Chem3D and **C**) optimized molecule with Avogadro.

**Table S1.** Characteristics of FEP-Druggable Pockets in the PonA1 Receptor

| Pockets | Vol. Hull* | Hydroph. Kyte* | Polar Res.* | Aromatic Res.* | Otyr atom | Nb. Res.* | Drugg Prob* | Standard Deviation |
| --- | --- | --- | --- | --- | --- | --- | --- | --- |
| P 17 | 455.24 | 1.5 | 0.25 | 0.17 | 0 | 12 | 0.99 | 0 |
| P 7 | 470.07 | 1.5 | 0.25 | 0.17 | 0 | 12 | 0.99 | 0 |
| P 22 | 337.6 | 1.17 | 0.38 | 0.15 | 0.04 | 13 | 0.99 | 0.01 |
| P 6 | 417.71 | 0.77 | 0.47 | 0.27 | 0.04 | 15 | 0.98 | 0 |
| P 24 | 540.25 | 1.1 | 0.4 | 0 | 0 | 10 | 0.96 | 0.01 |
| P 9 | 506.71 | 0.47 | 0.53 | 0.13 | 0 | 15 | 0.91 | 0.01 |
| P 3 | 473.5 | 0.24 | 0.5 | 0.21 | 0 | 14 | 0.89 | 0.03 |
| P 4 | 449.6 | 0.24 | 0.5 | 0.21 | 0 | 14 | 0.89 | 0.03 |
| P 10 | 547.61 | 0.16 | 0.56 | 0.13 | 0 | 16 | 0.83 | 0.01 |

*Vol. Hull* = Volume Hull; Hydrophob. Kyte*= Hydrophobic Kyte; Polar Res. *= Polar Residue Proportion; Aromatic Res. *= Aromatic Residue Proportion; Drugg Prob*= Druggability Probability; Nb. Res. * = Number of pocket residues.*

**Table S2.** Docking affinity of the FEP with the druggable pockets of the receptor‒protein complex.

| **Affinity (kcal mol^-1^)** | **Pocket** | **Mode** |
| --- | --- | --- |
| -10.7 | 10 | 1 |
| -10.4 | 4 | 7 |
| -10.4 | 24 | 1 |
| -9.5 | 6 | 1 |
| -9.4 | 9 | 1 |
| -9.1 | 17 | 1 |
| -8.6 | 3 | 1 |
| -8.4 | 7 | 2 |
| -8.2 | 22 | 2 |
